# Beyond Traditional Restorations: Management With Endocrown in a Late Adolescent

**DOI:** 10.7759/cureus.74394

**Published:** 2024-11-25

**Authors:** Abdulaziz Binrayes, Abdullatif A AlGhazzi, Saud M Alotaibi

**Affiliations:** 1 Dentistry, Vision College, Riyadh, SAU; 2 Prosthetic Dental Sciences, College of Dentistry, King Saud University, Riyadh, SAU; 3 Dentistry, College of Dentistry, King Saud University, Riyadh, SAU

**Keywords:** adolescence, endocrown, indirect restoration, prosthodontics, restoration

## Abstract

Endodontically treated molar rehabilitation is still challenging. Molars lose their mechanical properties after endodontic therapy. As a result of the pulp and surrounding dentin tissues being removed, they actually became brittle. A single one-piece restoration called an endodontic crown might be a viable alternative for crown restoration on molars with huge coronal damage presenting difficulties for endodontic treated teeth. This case report explains the successful restoration of an extensively damaged molar in a young individual using an endocrown. The decision to use this technique was guided by the patient’s age, the desire to maintain natural tooth structure, and the restrictions of traditional post-and-core restorations. The endocrown, fabricated from lithium disilicate glass, offered advantages such as minimal tooth preparation, a simplified restoration process, and extraordinary esthetic and functional outcomes. Long-term research is still required, but this particular case strengthens the argument for endocrowns as a beneficial restorative alternative, especially when more conventional methods could jeopardize the tooth's long-term health.

## Introduction

While many dentists are optimistic about the rehabilitation of teeth with considerable coronal destruction that have undergone endodontic treatment, a significant obstacle remains to be overcome. Retention and resistance are biomechanical concepts that diminish over time [1]. Clinicians plan restorative treatments based on the degree of dental tissue destruction and biomechanical changes induced by root canal therapy [2]. Restoring endodontically treated teeth with extensive coronal destruction is a major challenge in dentistry. Typically, such teeth are restored using full-coverage crowns supported by metal or fiber posts. However, this approach requires the removal of healthy tooth structure, potentially weakening the tooth and increasing the risk of root fractures [3].

The advent of adhesive dentistry and computer-aided design/computer-aided manufacturing (CAD/CAM) technology has introduced less-invasive restorative techniques, including endocrowns, a monolithic restoration that anchors to the pulp chamber for retention, thereby preserving natural tooth structure and minimizing the need for posts. This approach aligns with the principles of minimally invasive dentistry, which emphasizes the conservation of healthy tooth tissue [4]. The term "endocrown" refers to a single-piece ceramic construction introduced by Bindl and Mörmann in 1999 as an alternative to the full post-and-core-supported crown. To enhance macromechanical retention, this crown is affixed to the interior walls of the pulp chamber and the cavity borders; the application of adhesive cementation enhances micro-retention [5]. Endocrowns offer a robust and long-lasting restoration since they are usually constructed from a single block of ceramic or composite material. By properly bonding to the residual tooth structure using contemporary adhesive techniques, endocrowns improve retention and lower the chance of microleakage. The pulp chamber is used by endocrowns for extra retention. The pulp chamber's depth and form give the restoration a solid foundation, which promotes more uniform occlusal force distribution. Compared to regular crowns, an endocrown requires less invasive preparation. This increases the tooth's overall strength and resilience by preserving more of the tooth's original structure [6]. Due to the limitations associated with intra-radicular posts such as calcified root canals, narrow canals, or instrument fractures, dentists have increasingly considered alternatives like endocrowns and adhesive endodontic crowns. In addition, the limited interocclusal space makes it difficult to achieve adequate material thickness for conventional crowns [5-7].

Endocrowns offer a simpler and more direct alternative to traditional post-and-core restorations. They provide improved aesthetic qualities while requiring less time and expense. Additionally, the adhesive approach employed in endocrowns inhibits the penetration of bacteria from the crown toward the root and reduces marginal leakage [7]. Clinical studies have demonstrated excellent results with endocrowns, showing survival rates comparable to those of traditional crowns, particularly in molars [8]. Furthermore, several studies indicate that endocrowns have a lower rate of catastrophic failures, such as those requiring tooth extraction [7], as well as better stress distribution and higher fracture resistance than traditional crowns. Endocrowns are indicated in cases with substantial loss of tooth structure, restricted interocclusal space, or a short clinical crown, as they offer several advantages, such as simplified preparation, improved aesthetics, and reduced chair time, especially due to the possibility of single-visit fabrication using CAD/CAM technology [5-8].

Despite these benefits, the long-term clinical performance of endocrowns remains unclear [5-9]. Additional clinical studies with extended follow-up periods are necessary to provide definitive evidence for their widespread use. This case report aims to contribute to the existing literature by presenting the successful restoration of an endodontically treated molar with extensive coronal destruction using an endocrown in a young adult.

## Case presentation

**Figure 1:**
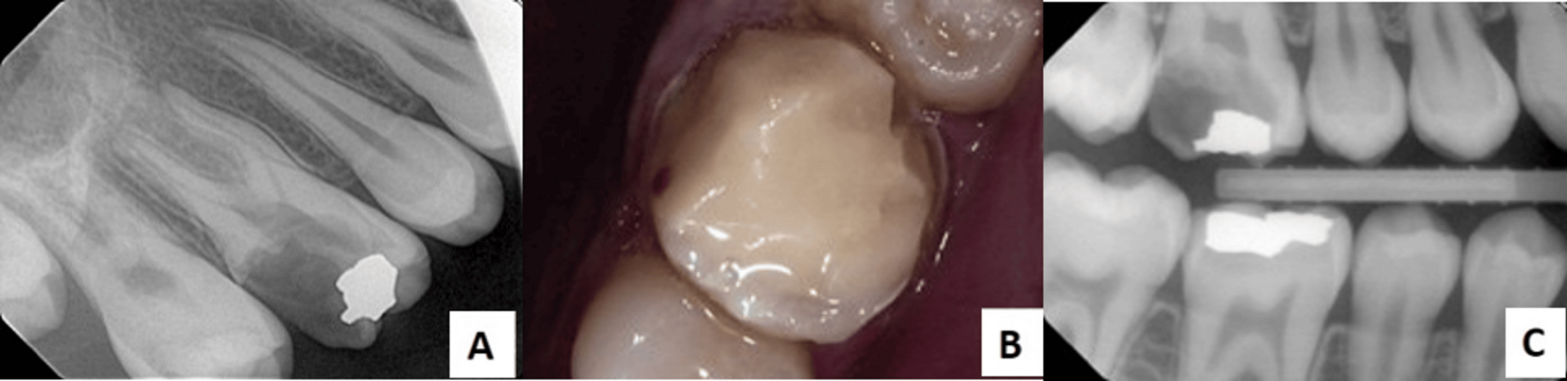
(A) Preoperative periapical radiograph; (B) Preoperative intraoral view with GIC restoration; (C) Preoperative bitewing radiograph. GIC: glass ionomer cement

A 15-year-old boy presented to the endodontic department of King Saud University Dental Hospital with pain in the upper right molar area. The patient reported no medical problems or medications. Upon clinical and radiographic examination (Figure 1), the upper right first molar was found to be extensively damaged with a periapical lesion associated with the root. Endodontic examinations revealed symptomatic irreversible pulpitis and symptomatic apical periodontitis. Although the tooth was restorable, the extent of tooth destruction rendered the prognosis uncertain. The patient’s father was presented with multiple treatment options: the first option involved a post-and-core followed by crown lengthening and a conventional crown; the second option, which had a better prognosis, was to extract the tooth and restore it with an implant-supported prosthesis. However, The first option was excluded because of the lengthy procedure the father's wish to avoid any surgical procedures, and the second option was not feasible at that time due to the patient’s age. The last option was the provision of an endocrown to maintain the tooth until the patient reached the appropriate age for a dental implant if needed. Consequently, the patient was referred to the endodontic department for root canal treatment and subsequently referred back for the fabrication for the final restoration. 

Before beginning the procedure, the teeth were examined for shade selection from various angles and distances while they were wet using the Vita traditional shade guide (Vita Zahnfabrik, Bad Sackingen, Germany). Images of the tooth with and without the shade were captured and communicated to the laboratory and A2 was the final shade chosen. The preparation involved reducing undercuts and making occlusogingival height adjustments to achieve occlusal clearance. Wheel burs and green tapered diamonds were utilized in the preparation procedure. The finish line for endocrown preparation was a butt joint of a 90-degree band of enamel approximately 1-2 mm in width. The pulp chamber depth was greater than 3 mm and minimal undercuts were noticed in the pulp chamber which were managed by holding the diamond tapered bur 7-degree occlusal taper to the long axis of the tooth. 

**Figure 2:**
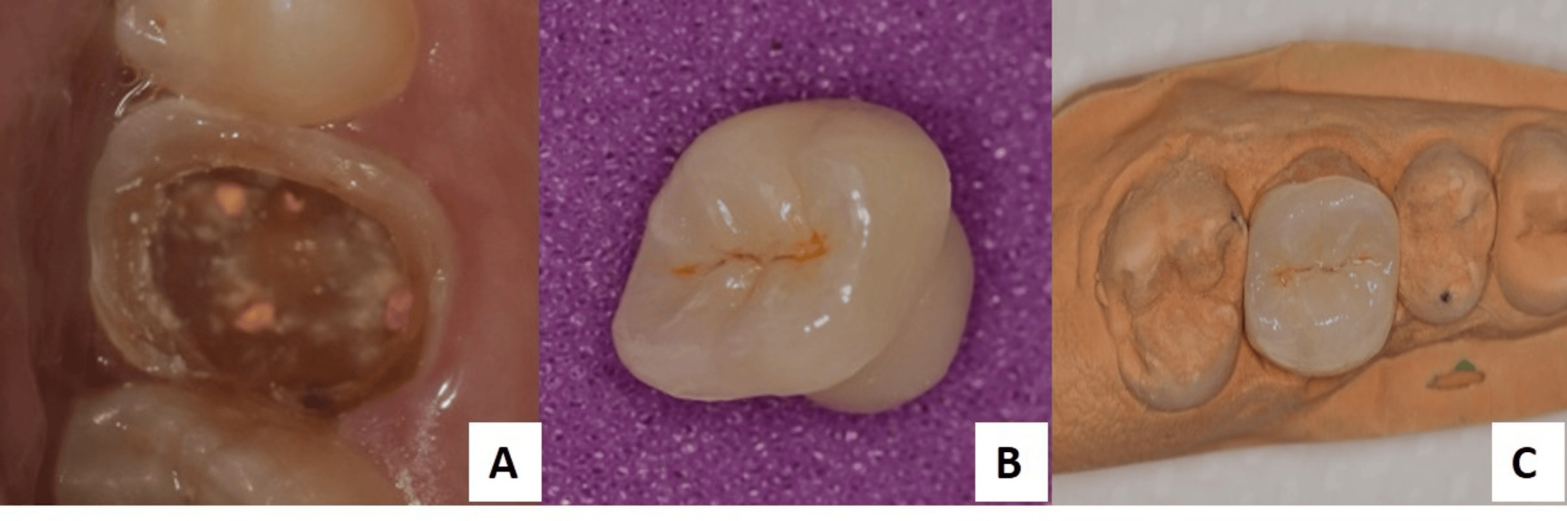
(A) Intraoral view after crown preparation and before sealing of orifices with flowable composites; (B) Lithium disilicate endocrown; (C) Crown fitted on master-cast.

The preparation was performed according to the guidelines published by the Canadian Dental Association (Figure 2). After preparation, acid etchant (3M™ Scotchbond™ Universal Etchant Etching Gel (32% phosphoric acid); 3M Company, Saint Paul, Minnesota, United States) was applied for 15 seconds and rinsed for another 15 seconds and dried. Followed by bonding (3M Scotchbond Universal Adhesive; 3M Company) applied and air-blown for five seconds and light cured for 20 seconds. Flowable composite shade A2 (3M ESPE™ Filtek™ Ultimate Universal; 3M Company) was used to block the orifices and light cured for 40 seconds. After preparation, polyvinyl siloxane impressions were obtained using regular and light bodies (3M Express XT Regular Body and Light Body; 3M Company). A heat press technique was used to fabricate the lithium disilicate (IPS Emax Press; Ivoclar Vivadent, AG Schaan, Liechtenstein) endocrown. A cast was created from the polyvinyl siloxane (PVS) impression, and a wax pattern was then made to create a mold. Finally, a ceramic ingot was heat pressed into the mold to create the final restoration.

**Figure 3:**
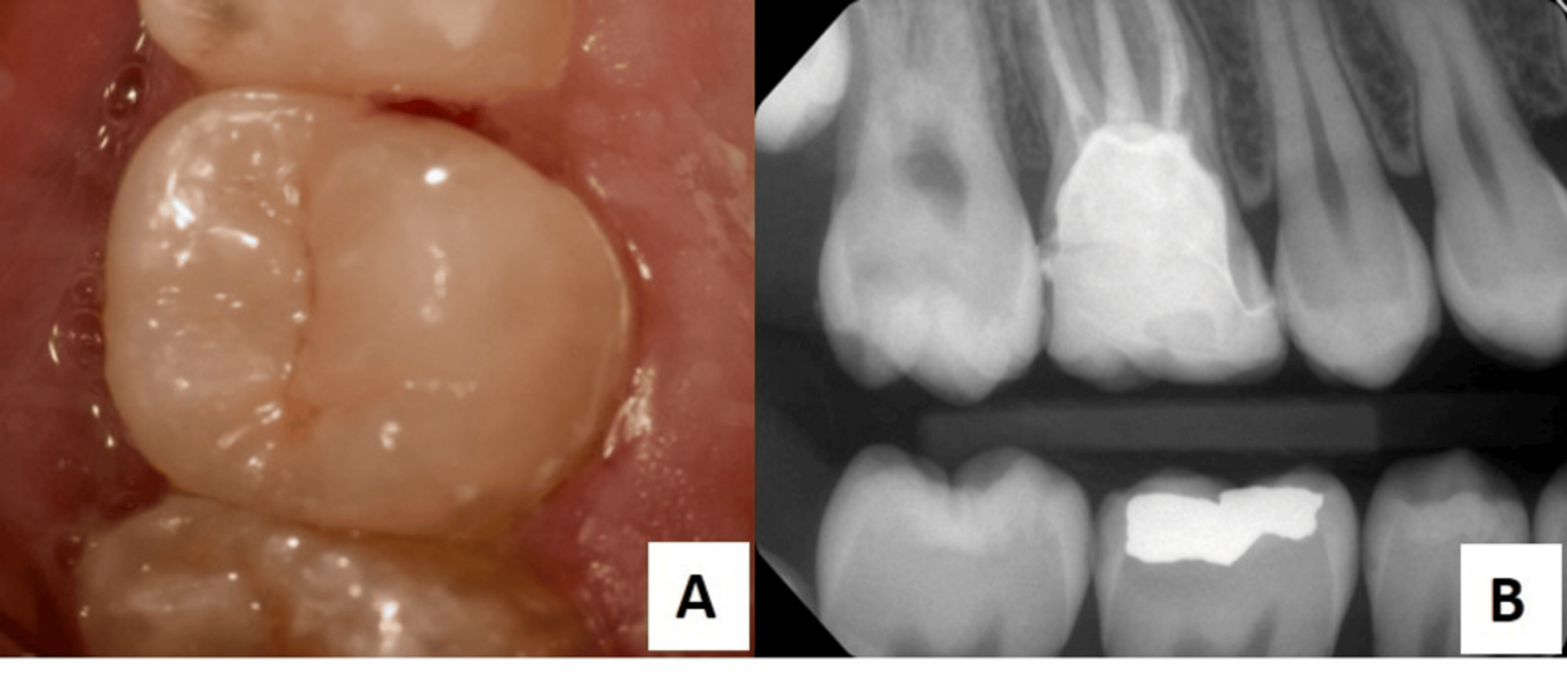
(A) Post-cementation intraoral photo; (B) Post-cementation bitewing radiograph.

The process of how the endocrown was cemented is described here. The old restoration was removed carefully without any damage to the remaining tooth structure and the surface and crown were cleaned. Buffered hydrofluoric acid gel (9.5%) (BISCO, Inc., Schaumburg, Illinois, United States) was applied for 20 seconds and then rinsed with copious amounts of water and air dried. A porcelain silane coupling agent (Bis-Silane Porcelain Primer; BISCO, Inc.) was applied following the manufacturer's instructions. The two-part silane coupling agent was mixed in a ratio of 1:1. The first coat was applied to the etched intaglio surface of the endocrown for one minute, followed by a second coat, which was left in the cavity for 30 seconds and after that gently dried using an air syringe. A self-etch/self-adhesive dual-cure cement, Maxcem Elite™ Universal Resin Cement (KaVo Kerr, Brea, California, United States) was applied to the tooth pulp chamber surface and the intaglio surface of the endocrown, and the endocrown was seated over the prepared teeth. Excess cement was removed, and the cement was cured for 20 seconds on each surface (occlusal, buccal, and palatal). A radiograph was taken to confirm the removal of any remnants of cement (Figure 3).

Occlusion was checked before and after cementation with articulating paper to determine any unwanted areas of occlusion in centric and eccentric. Bausch Arti Fol Metallic Shimstock Flim 12µ (Dr. Jean Bausch GmbH & Co. KG, Köln, Germany) was used to evaluate the amount of occlusion on all the teeth and verified that endocrown was within centric occlusion. The patient was followed up after one week of endocrown cementation and the endocrown was checked for its integrity and no problems were discovered.

**Figure 4:**
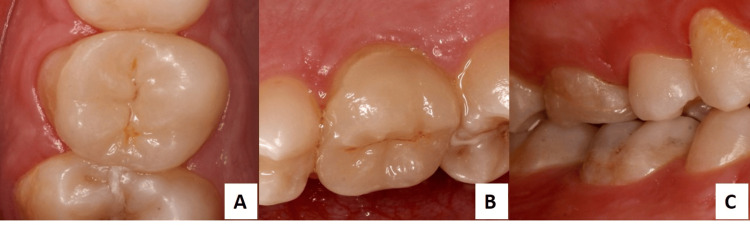
(A) Two-year follow-up occlusal view; (B) Follow-up palatal view; (C) Follow-up buccal view.

**Figure 5:**
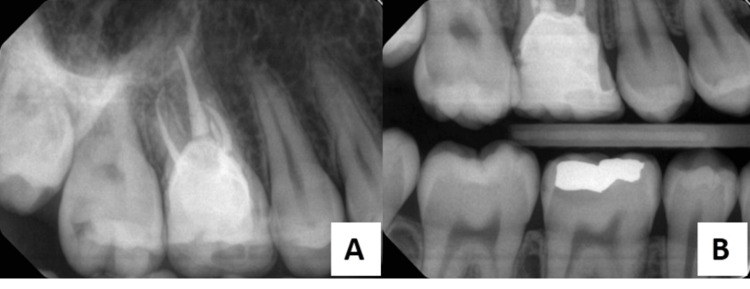
(A) Two-year follow-up periapical radiograph; (B) Two-year follow-up bitewing radiograph.

The patient was referred for a follow-up appointment approximately two years later (Figures 4, 5). At that time, the patient presented with healthy gingiva and sound tooth structure in the upper right first molar, as well as a healed periapical area. Further follow-up appointments were scheduled.

## Discussion

Careful preparation is necessary to address the clinical challenges of restoring molars with substantial coronal damage. Dentists must select the optimal treatment strategy to ensure effective results and the long-term durability of the affected molars [10]. While all molars can benefit from an endocrown, those with narrow canals, calcified root canals, limited inter-arch space, or clinically low crowns will benefit the most. However, this is not recommended if the cervical margin measures less than 2 mm along most of its circumference, if adhesion cannot be guaranteed, or if the pulpal chamber depth is less than 3 mm [5-9].

The successful restoration of a severely damaged molar in a young patient using an endocrown, as shown in this case report, highlights the advantages of this technique as an alternative to traditional post-and-core restorations. The decision to use an endocrown was influenced by the patient’s age, the desire to retain the natural tooth, and the limitations of post-and-core restorations in this case. The preparation aimed to create a surface sufficiently sturdy and broad to withstand the compressive forces prevalent in molars. The prepared surface was aligned parallel to the occlusal surface of the tooth to provide stress resistance along its long axis. Compared to teeth with prosthetic crowns, endocrowns experience less stress [6,11].

Endocrowns provide several benefits consistent with the principles of minimally invasive dentistry. Compared to conventional crowns, they require less removal of sound tooth structures as they use the pulp chamber for retention [7], thereby preserving the tooth's structural integrity and reducing the risk of fracture. This consideration is particularly crucial for younger patients, as excessive tooth preparation can negatively affect the long-term prognosis [12] of the tooth and shorten chair time by simplifying the restorative procedure. This can improve patient satisfaction and comfort, especially for young people who might be hesitant to undergo major dental procedures [6]. Another important consideration is that the requirement for macroretentive crown preparation has diminished as a result of the development of new adhesive cementation techniques relying on chemical adhesion and micromechanical retention [13]. The preparation technique requires a high level of skill and precision. Many clinicians may not feel confident in performing this procedure due to its complexity. Achieving the correct preparation is crucial. The tooth must be reduced to create a flat surface with a uniform cervical margin, which can be difficult, especially in teeth with irregular shapes [12-14]. In the present case, for occlusal clearance, the preparation entailed lowering undercuts and adjusting occlusogingival height. The orifices of the canals were sealed by flowable composite.

Endocrowns can be fabricated using heat pressing or CAD/CAM technology [7]. The heat pressing technique involves creating a cast from a traditional imprint using polyether or polyvinyl siloxane. A wax pattern is subsequently created and burned to create a refractory mold, into which ceramic ingots are heat pressed in a ceramic furnace. This procedure offers benefits such as ease of use, speed, and proper fit for the restoration [8,9]. During the CAD/CAM process, a scanner captures a digital impression of a tooth, either from a traditional imprint or directly from the tooth itself. CAD software is then used to design the restoration, which is milled from a ceramic block. This technology allows for high-quality restorations in a single session, eliminating the need for diagnostic wax-ups and allowing for the selection of anatomical features [14], while optimizing data storage capabilities. In the present case, successful restoration was aided by the use of lithium disilicate glass in the endocrown production process. Due to its superior mechanical properties, including high flexural strength and fracture toughness, lithium disilicate is suitable for restoring posterior teeth subjected to high occlusal forces. Moreover, its exceptional aesthetics facilitate a smooth transition into the natural dentition [15]. The high performance of lithium disilicate endocrowns was also reported by Altier et al., who found that lithium disilicate ceramic endocrowns showed higher fracture strength than indirect resin composites [16]. Additionally, lithium disilicate-based ceramic endocrowns are highly effective due to their adhesive properties and micromechanical interlocking with resin cement [13].

In 2018, Dartora et al. evaluated endodontically treated teeth restored with endocrowns and found that a 5 mm extension provided better mechanical performance, with lower intensity and better stress distribution [17]. Taha et al.'s in vitro study demonstrated that endocrowns with axial reduction and a shoulder finish line exhibited higher fracture resistance values than those with a butt margin design [18]. Biacchi et al. compared conventional crowns and endocrowns, finding endocrowns more resistant to compressive forces and highlighting their role in stress distribution [19]. Schultheis et al. suggested that endocrowns are a more reliable option for posterior load-bearing teeth, whereas a bilayer configuration is more susceptible to reducing load fracture failure [20].

Although research on the long-term clinical performance of endocrowns is ongoing, preliminary data indicate promising results, with survival rates comparable to those of conventional crowns, particularly in molars [5-9,11-20]. Nevertheless, additional studies with longer follow-up periods are required to conclusively determine their effectiveness and durability. This case report contributes to the increasing body of research showing that endocrowns can be used to restore teeth with substantial coronal damage that have undergone endodontic treatment. This illustrates how the method can maintain tooth structure, simplify the restorative procedure, and produce acceptable functional and aesthetic results. It is anticipated that endocrowns, as minimally invasive and successful restorative alternatives, will receive more attention in adhesive dentistry and CAD/CAM technologies.

## Conclusions

This case report demonstrates the successful application of endocrowns in a challenging scenario involving a young patient with extensive tooth damage. Using this minimally invasive technique, we preserved the natural tooth structure and achieved favorable outcomes. While long-term studies are needed, this case adds to the growing evidence supporting endocrowns as a valuable restorative option, particularly in cases where traditional approaches may compromise future tooth health. It is imperative to emphasize that a cautious case selection process and meticulous application of endocrown and adhesive procedures are critical for the success of this treatment modality.
